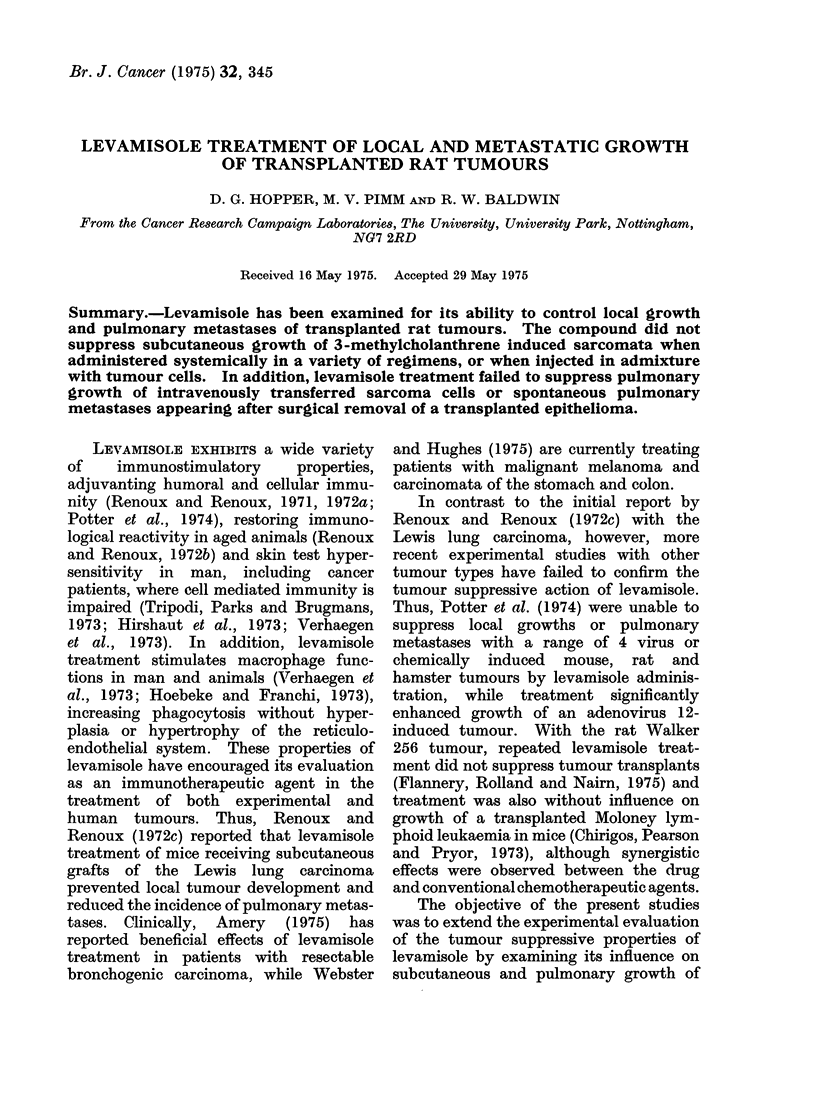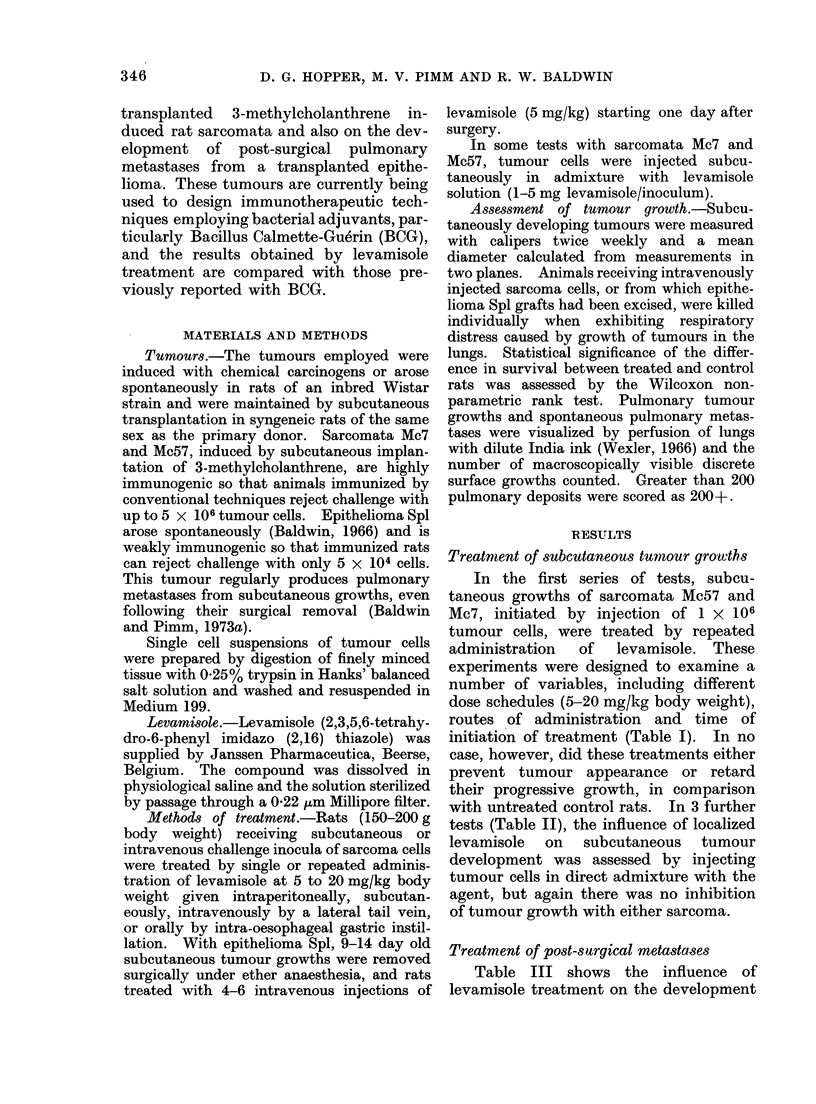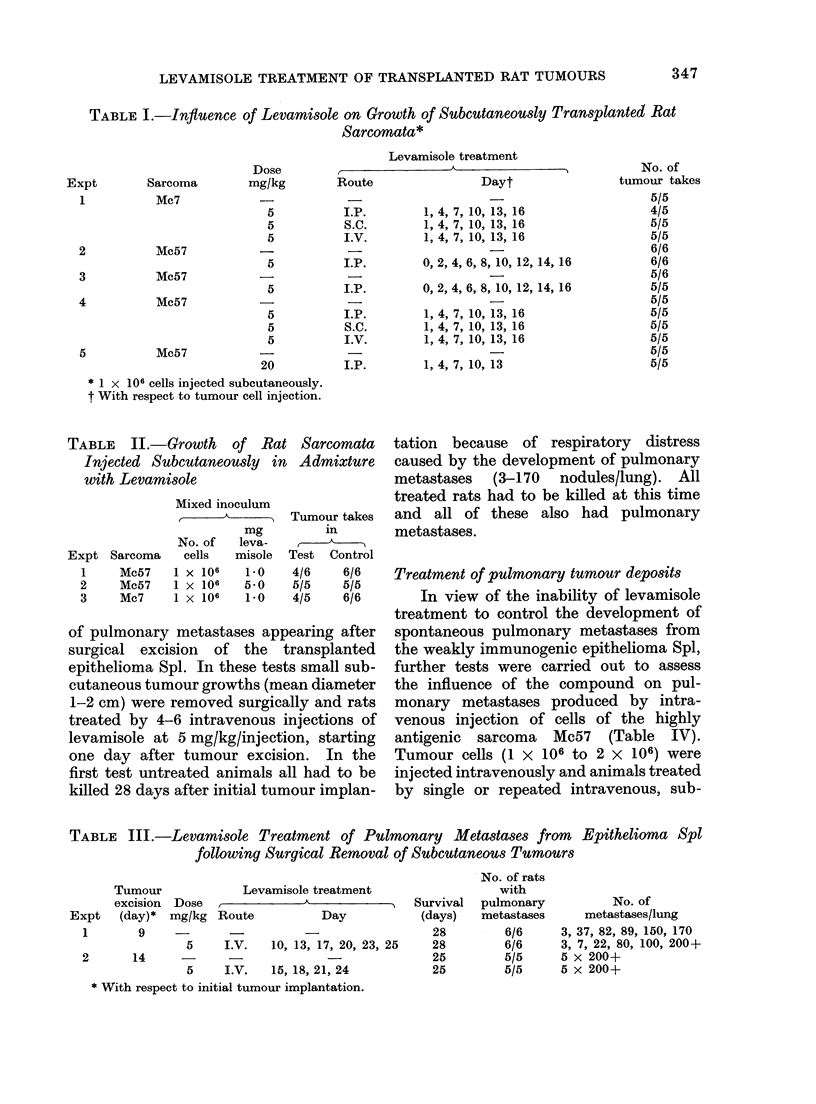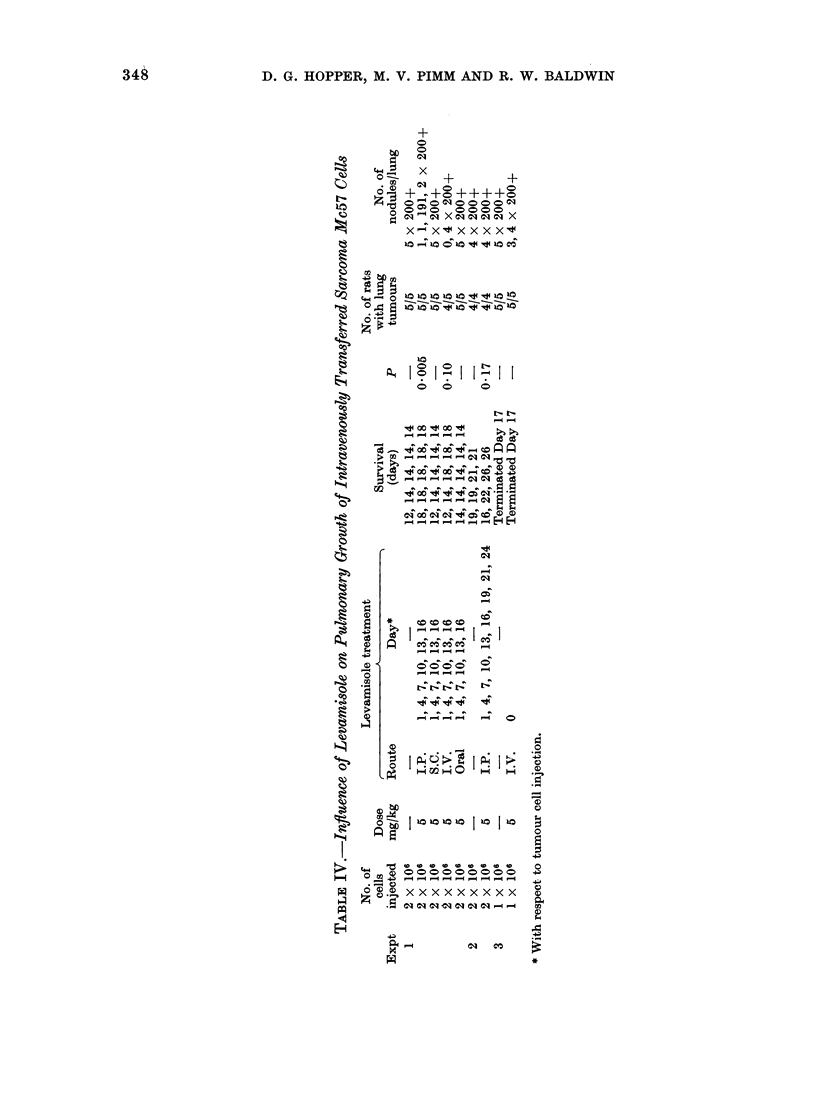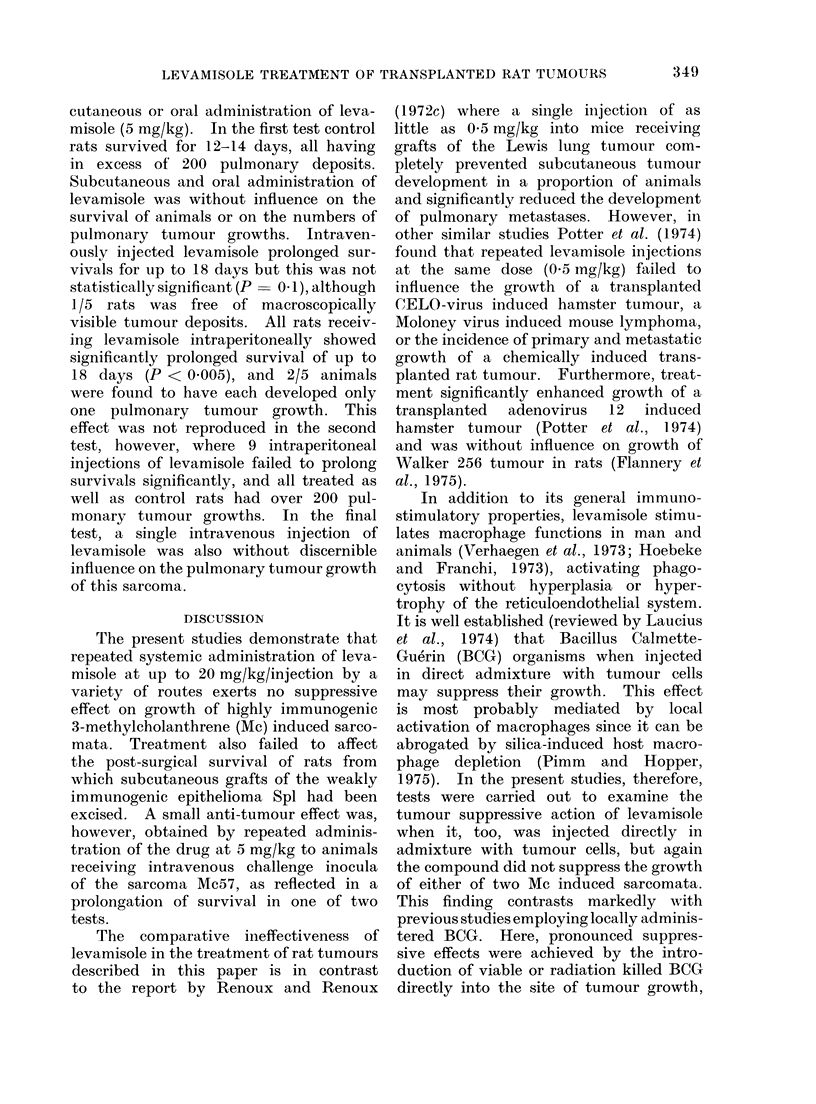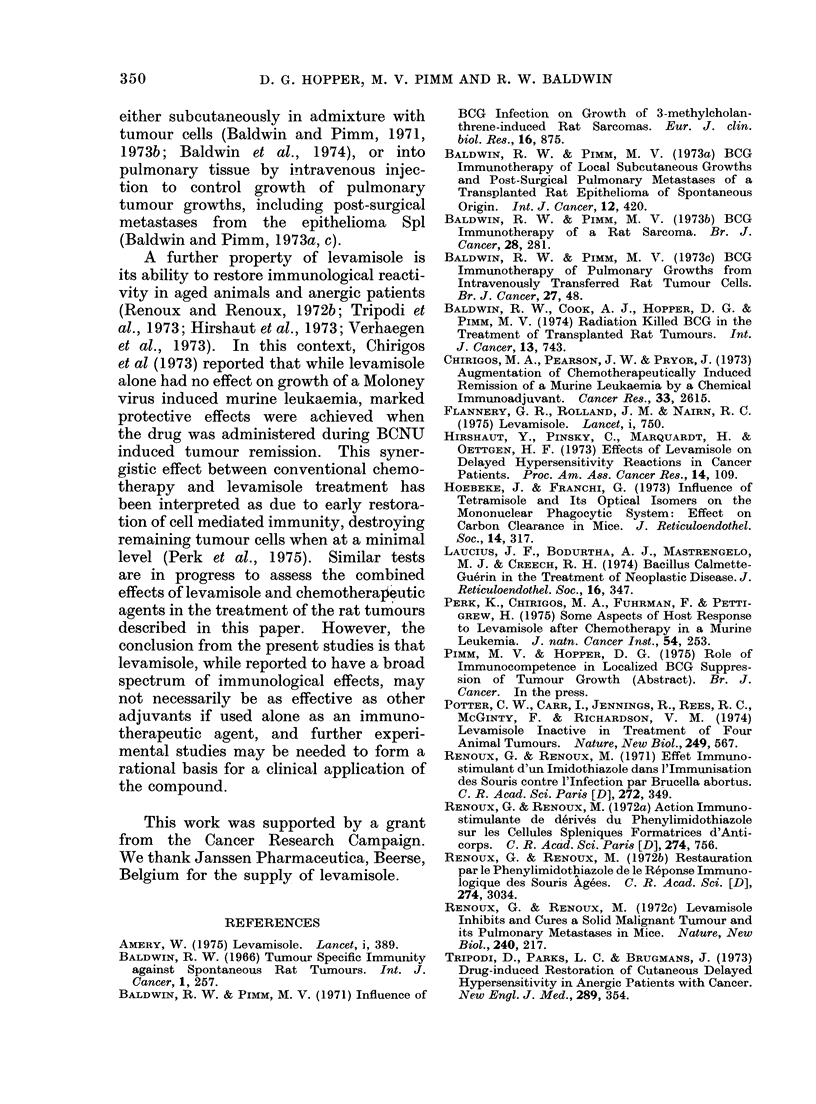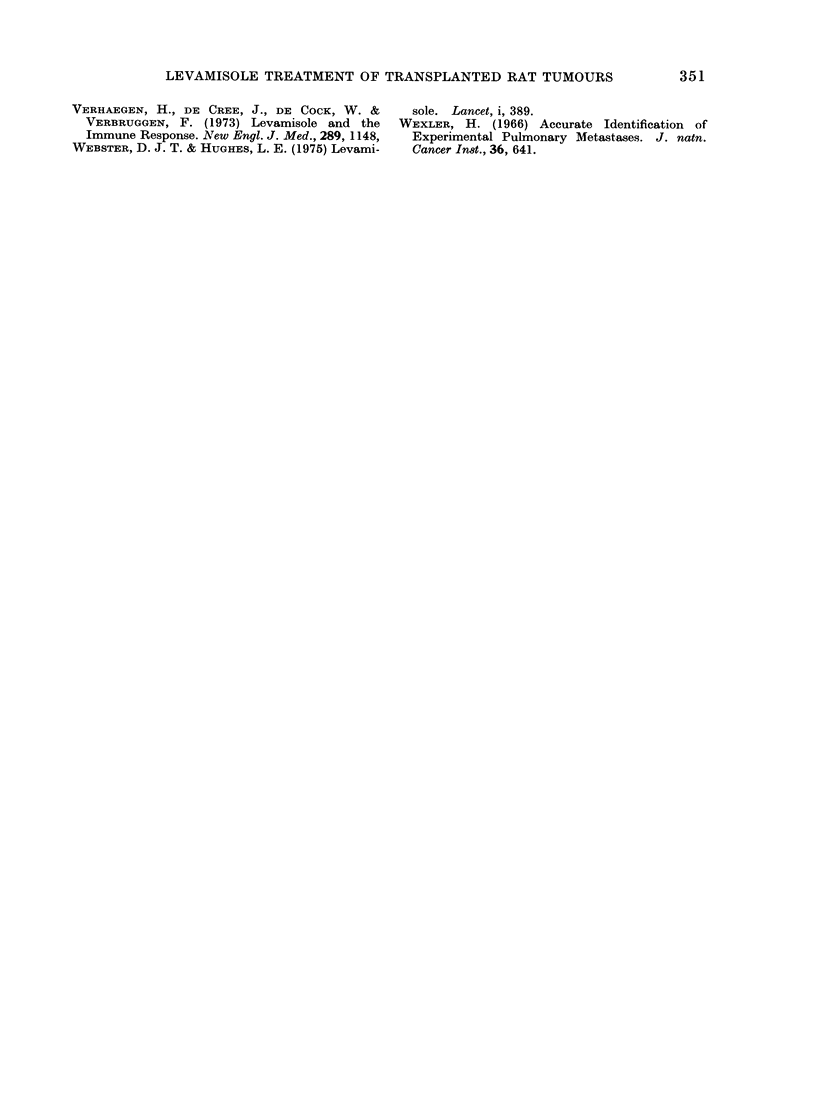# Levamisole treatment of local and metastatic growth of transplanted rat tumours.

**DOI:** 10.1038/bjc.1975.233

**Published:** 1975-09

**Authors:** D. G. Hopper, M. V. Pimm, R. W. Baldwin

## Abstract

Levamisole has been examined for its ability to control local growth and pulmonary metastases of transplanted rat tumours. The compound did not suppress subcutaneous growth of 3-methylcholanthrene induced sarcomata when administered systemically in a variety of regimens, or when injected in admixture with tumour cells. In addition, levamisole treatment failed to suppress pulmonary growth of intravenously transferred sarcoma cells or spontaneous pulmonary metastases appearing after surgical removal of a transplanted epithelioma.


*Br. J. Cancer* (1975) **32**, 345

### LEVAMISOLE TREATMENT OF LOCAL AND METASTATIC GROWTH OF TRANSPLANTED RAT TUMOURS

D. G. HOPPER, M. V. PIMM AND R. W. BALDWIN

*From the Cancer Research Campaign Laboratories, The University, University Park, Nottingham, NG7 2RD*

Received 16 May 1975. Accepted 29 May 1975

**Summary.—Levamisole has been examined for its ability to control local growth and pulmonary metastases of transplanted rat tumours. The compound did not suppress subcutaneous growth of 3-methylcholanthrene induced sarcomata when administered systemically in a variety of regimens, or when injected in admixture with tumour cells. In addition, levamisole treatment failed to suppress pulmonary growth of intravenously transferred sarcoma cells or spontaneous pulmonary metastases appearing after surgical removal of a transplanted epithelioma.**

LEVAMISOLE EXHIBITS a wide variety of immunostimulatory properties, adjuvanting humoral and cellular immunity (Renoux and Renoux, 1971, 1972*a*; Potter *et al.*, 1974), restoring immunological reactivity in aged animals (Renoux and Renoux, 1972*b*) and skin test hypersensitivity in man, including cancer patients, where cell mediated immunity is impaired (Tripodi, Parks and Brugmans, 1973; Hirshaut *et al.*, 1973; Verhaegen *et al.*, 1973). In addition, levamisole treatment stimulates macrophage functions in man and animals (Verhaegen *et al.*, 1973; Hoebeke and Franchi, 1973), increasing phagocytosis without hyperplasia or hypertrophy of the reticuloendothelial system. These properties of levamisole have encouraged its evaluation as an immunotherapeutic agent in the treatment of both experimental and human tumours. Thus, Renoux and Renoux (1972*c*) reported that levamisole treatment of mice receiving subcutaneous grafts of the Lewis lung carcinoma prevented local tumour development and reduced the incidence of pulmonary metastases. Clinically, Amery (1975) has reported beneficial effects of levamisole treatment in patients with resectable bronchogenic carcinoma, while Webster and Hughes (1975) are currently treating patients with malignant melanoma and carcinomata of the stomach and colon.

In contrast to the initial report by Renoux and Renoux (1972*c*) with the Lewis lung carcinoma, however, more recent experimental studies with other tumour types have failed to confirm the tumour suppressive action of levamisole. Thus, Potter *et al.* (1974) were unable to suppress local growths or pulmonary metastases with a range of 4 virus or chemically induced mouse, rat and hamster tumours by levamisole administration, while treatment significantly enhanced growth of an adenovirus 12-induced tumour. With the rat Walker 256 tumour, repeated levamisole treatment did not suppress tumour transplants (Flannery, Rolland and Nairn, 1975) and treatment was also without influence on growth of a transplanted Moloney lymphoid leukaemia in mice (Chirigos, Pearson and Pryor, 1973), although synergistic effects were observed between the drug and conventional chemotherapeutic agents.

The objective of the present studies was to extend the experimental evaluation of the tumour suppressive properties of levamisole by examining its influence on subcutaneous and pulmonary growth of transplanted 3-methylcholanthrene induced rat sarcomata and also on the development of post-surgical pulmonary metastases from a transplanted epithelioma. These tumours are currently being used to design immunotherapeutic techniques employing bacterial adjuvants, particularly Bacillus Calmette-Guérin (BCG), and the results obtained by levamisole treatment are compared with those previously reported with BCG.

### MATERIALS AND METHODS

*Tumours.*—The tumours employed were induced with chemical carcinogens or arose spontaneously in rats of an inbred Wistar strain and were maintained by subcutaneous transplantation in syngeneic rats of the same sex as the primary donor. Sarcomata Mc7 and Mc57, induced by subcutaneous implantation of 3-methylcholanthrene, are highly immunogenic so that animals immunized by conventional techniques reject challenge with up to $5 \times 10^6$ tumour cells. Epithelioma Spl arose spontaneously (Baldwin, 1966) and is weakly immunogenic so that immunized rats can reject challenge with only $5 \times 10^4$ cells. This tumour regularly produces pulmonary metastases from subcutaneous growths, even following their surgical removal (Baldwin and Pimm, 1973*a*).

Single cell suspensions of tumour cells were prepared by digestion of finely minced tissue with 0·25% trypsin in Hanks' balanced salt solution and washed and resuspended in Medium 199.

*Levamisole.*—Levamisole (2,3,5,6-tetrahydro-6-phenyl imidazo (2,16) thiazole) was supplied by Janssen Pharmaceutica, Beerse, Belgium. The compound was dissolved in physiological saline and the solution sterilized by passage through a 0·22 μm Millipore filter.

*Methods of treatment.*—Rats (150–200 g body weight) receiving subcutaneous or intravenous challenge inocula of sarcoma cells were treated by single or repeated administration of levamisole at 5 to 20 mg/kg body weight given intraperitoneally, subcutaneously, intravenously by a lateral tail vein, or orally by intra-oesophageal gastric instillation. With epithelioma Spl, 9–14 day old subcutaneous tumour growths were removed surgically under ether anaesthesia, and rats treated with 4–6 intravenous injections of levamisole (5 mg/kg) starting one day after surgery.

In some tests with sarcomata Mc7 and Mc57, tumour cells were injected subcutaneously in admixture with levamisole solution (1–5 mg levamisole/inoculum).

*Assessment of tumour growth.*—Subcutaneously developing tumours were measured with calipers twice weekly and a mean diameter calculated from measurements in two planes. Animals receiving intravenously injected sarcoma cells, or from which epithelioma Spl grafts had been excised, were killed individually when exhibiting respiratory distress caused by growth of tumours in the lungs. Statistical significance of the difference in survival between treated and control rats was assessed by the Wilcoxon nonparametric rank test. Pulmonary tumour growths and spontaneous pulmonary metastases were visualized by perfusion of lungs with dilute India ink (Wexler, 1966) and the number of macroscopically visible discrete surface growths counted. Greater than 200 pulmonary deposits were scored as 200+.

### RESULTS

### *Treatment of subcutaneous tumour growths*

In the first series of tests, subcutaneous growths of sarcomata Mc57 and Mc7, initiated by injection of $1 \times 10^6$ tumour cells, were treated by repeated administration of levamisole. These experiments were designed to examine a number of variables, including different dose schedules (5–20 mg/kg body weight), routes of administration and time of initiation of treatment (Table I). In no case, however, did these treatments either prevent tumour appearance or retard their progressive growth, in comparison with untreated control rats. In 3 further tests (Table II), the influence of localized levamisole on subcutaneous tumour development was assessed by injecting tumour cells in direct admixture with the agent, but again there was no inhibition of tumour growth with either sarcoma.

### *Treatment of post-surgical metastases*

Table III shows the influence of levamisole treatment on the development

TABLE I.—*Influence of Levamisole on Growth of Subcutaneously Transplanted Rat Sarcomata**

| Expt | Sarcoma | Dose mg/kg | Levamisole treatment: Route | Levamisole treatment: Day† | No. of tumour takes |
|---|---|---|---|---|---|
| 1 | Mc7 | — | — | — | 5/5 |
| | | 5 | I.P. | 1, 4, 7, 10, 13, 16 | 4/5 |
| | | 5 | S.C. | 1, 4, 7, 10, 13, 16 | 5/5 |
| | | 5 | I.V. | 1, 4, 7, 10, 13, 16 | 5/5 |
| 2 | Mc57 | — | — | — | 6/6 |
| | | 5 | I.P. | 0, 2, 4, 6, 8, 10, 12, 14, 16 | 6/6 |
| 3 | Mc57 | — | — | — | 5/6 |
| | | 5 | I.P. | 0, 2, 4, 6, 8, 10, 12, 14, 16 | 5/5 |
| 4 | Mc57 | — | — | — | 5/5 |
| | | 5 | I.P. | 1, 4, 7, 10, 13, 16 | 5/5 |
| | | 5 | S.C. | 1, 4, 7, 10, 13, 16 | 5/5 |
| | | 5 | I.V. | 1, 4, 7, 10, 13, 16 | 5/5 |
| 5 | Mc57 | — | — | — | 5/5 |
| | | 20 | I.P. | 1, 4, 7, 10, 13 | 5/5 |

* 1 × $10^6$ cells injected subcutaneously.
† With respect to tumour cell injection.

TABLE II.—*Growth of Rat Sarcomata Injected Subcutaneously in Admixture with Levamisole*

| Expt | Sarcoma | Mixed inoculum: No. of cells | Mixed inoculum: mg levamisole | Tumour takes in: Test | Tumour takes in: Control |
|---|---|---|---|---|---|
| 1 | Mc57 | 1 × $10^6$ | 1·0 | 4/6 | 6/6 |
| 2 | Mc57 | 1 × $10^6$ | 5·0 | 5/5 | 5/5 |
| 3 | Mc7 | 1 × $10^6$ | 1·0 | 4/5 | 6/6 |

of pulmonary metastases appearing after surgical excision of the transplanted epithelioma Spl. In these tests small subcutaneous tumour growths (mean diameter 1–2 cm) were removed surgically and rats treated by 4–6 intravenous injections of levamisole at 5 mg/kg/injection, starting one day after tumour excision. In the first test untreated animals all had to be killed 28 days after initial tumour implantation because of respiratory distress caused by the development of pulmonary metastases (3–170 nodules/lung). All treated rats had to be killed at this time and all of these also had pulmonary metastases.

### *Treatment of pulmonary tumour deposits*

In view of the inability of levamisole treatment to control the development of spontaneous pulmonary metastases from the weakly immunogenic epithelioma Spl, further tests were carried out to assess the influence of the compound on pulmonary metastases produced by intravenous injection of cells of the highly antigenic sarcoma Mc57 (Table IV). Tumour cells (1 × $10^6$ to 2 × $10^6$) were injected intravenously and animals treated by single or repeated intravenous, sub-

TABLE III.—*Levamisole Treatment of Pulmonary Metastases from Epithelioma Spl following Surgical Removal of Subcutaneous Tumours*

| Expt | Tumour excision (day)* | Dose mg/kg | Levamisole treatment: Route | Levamisole treatment: Day | Survival (days) | No. of rats with pulmonary metastases | No. of metastases/lung |
|---|---|---|---|---|---|---|---|
| 1 | 9 | — | — | — | 28 | 6/6 | 3, 37, 82, 89, 150, 170 |
| | | 5 | I.V. | 10, 13, 17, 20, 23, 25 | 28 | 6/6 | 3, 7, 22, 80, 100, 200+ |
| 2 | 14 | — | — | — | 25 | 5/5 | 5 × 200+ |
| | | 5 | I.V. | 15, 18, 21, 24 | 25 | 5/5 | 5 × 200+ |

* With respect to initial tumour implantation.

TABLE IV.—*Influence of Levamisole on Pulmonary Growth of Intravenously Transferred Sarcoma Mc57 Cells*

| Expt | No. of cells injected | Dose mg/kg | Levamisole treatment: Route | Levamisole treatment: Day* | Survival (days) | $P$ | No. of rats with lung tumours | No. of nodules/lung |
|---|---|---|---|---|---|---|---|---|
| 1 | $2 \times 10^6$ | — | — | — | 12, 14, 14, 14, 14 | — | 5/5 | 5 × 200+ |
| | $2 \times 10^6$ | 5 | I.P. | 1, 4, 7, 10, 13, 16 | 18, 18, 18, 18, 18 | 0·005 | 5/5 | 1, 1, 191, 2 × 200+ |
| | $2 \times 10^6$ | 5 | S.C. | 1, 4, 7, 10, 13, 16 | 12, 14, 14, 14, 14 | — | 5/5 | 5 × 200+ |
| | $2 \times 10^6$ | 5 | I.V. | 1, 4, 7, 10, 13, 16 | 12, 14, 18, 18, 18 | 0·10 | 4/5 | 0, 4 × 200+ |
| | $2 \times 10^6$ | 5 | Oral | 1, 4, 7, 10, 13, 16 | 14, 14, 14, 14, 14 | — | 5/5 | 5 × 200+ |
| 2 | $2 \times 10^6$ | — | — | — | 19, 19, 21, 21 | — | 4/4 | 4 × 200+ |
| | $2 \times 10^6$ | 5 | I.P. | 1, 4, 7, 10, 13, 16, 19, 21, 24 | 16, 22, 26, 26 | 0·17 | 4/4 | 4 × 200+ |
| 3 | $1 \times 10^6$ | — | — | — | Terminated Day 17 | — | 5/5 | 5 × 200+ |
| | $1 \times 10^6$ | 5 | I.V. | 0 | Terminated Day 17 | — | 5/5 | 3, 4 × 200+ |

* With respect to tumour cell injection.

cutaneous or oral administration of levamisole (5 mg/kg). In the first test control rats survived for 12–14 days, all having in excess of 200 pulmonary deposits. Subcutaneous and oral administration of levamisole was without influence on the survival of animals or on the numbers of pulmonary tumour growths. Intravenously injected levamisole prolonged survivals for up to 18 days but this was not statistically significant ($P = 0{\cdot}1$), although 1/5 rats was free of macroscopically visible tumour deposits. All rats receiving levamisole intraperitoneally showed significantly prolonged survival of up to 18 days ($P < 0{\cdot}005$), and 2/5 animals were found to have each developed only one pulmonary tumour growth. This effect was not reproduced in the second test, however, where 9 intraperitoneal injections of levamisole failed to prolong survivals significantly, and all treated as well as control rats had over 200 pulmonary tumour growths. In the final test, a single intravenous injection of levamisole was also without discernible influence on the pulmonary tumour growth of this sarcoma.

### DISCUSSION

The present studies demonstrate that repeated systemic administration of levamisole at up to 20 mg/kg/injection by a variety of routes exerts no suppressive effect on growth of highly immunogenic 3-methylcholanthrene (Mc) induced sarcomata. Treatment also failed to affect the post-surgical survival of rats from which subcutaneous grafts of the weakly immunogenic epithelioma Spl had been excised. A small anti-tumour effect was, however, obtained by repeated administration of the drug at 5 mg/kg to animals receiving intravenous challenge inocula of the sarcoma Mc57, as reflected in a prolongation of survival in one of two tests.

The comparative ineffectiveness of levamisole in the treatment of rat tumours described in this paper is in contrast to the report by Renoux and Renoux (1972*c*) where a single injection of as little as 0·5 mg/kg into mice receiving grafts of the Lewis lung tumour completely prevented subcutaneous tumour development in a proportion of animals and significantly reduced the development of pulmonary metastases. However, in other similar studies Potter *et al.* (1974) found that repeated levamisole injections at the same dose (0·5 mg/kg) failed to influence the growth of a transplanted CELO-virus induced hamster tumour, a Moloney virus induced mouse lymphoma, or the incidence of primary and metastatic growth of a chemically induced transplanted rat tumour. Furthermore, treatment significantly enhanced growth of a transplanted adenovirus 12 induced hamster tumour (Potter *et al.*, 1974) and was without influence on growth of Walker 256 tumour in rats (Flannery *et al.*, 1975).

In addition to its general immunostimulatory properties, levamisole stimulates macrophage functions in man and animals (Verhaegen *et al.*, 1973; Hoebeke and Franchi, 1973), activating phagocytosis without hyperplasia or hypertrophy of the reticuloendothelial system. It is well established (reviewed by Laucius *et al.*, 1974) that Bacillus Calmette-Guérin (BCG) organisms when injected in direct admixture with tumour cells may suppress their growth. This effect is most probably mediated by local activation of macrophages since it can be abrogated by silica-induced host macrophage depletion (Pimm and Hopper, 1975). In the present studies, therefore, tests were carried out to examine the tumour suppressive action of levamisole when it, too, was injected directly in admixture with tumour cells, but again the compound did not suppress the growth of either of two Mc induced sarcomata. This finding contrasts markedly with previous studies employing locally administered BCG. Here, pronounced suppressive effects were achieved by the introduction of viable or radiation killed BCG directly into the site of tumour growth, either subcutaneously in admixture with tumour cells (Baldwin and Pimm, 1971, 1973*b*; Baldwin *et al.*, 1974), or into pulmonary tissue by intravenous injection to control growth of pulmonary tumour growths, including post-surgical metastases from the epithelioma Spl (Baldwin and Pimm, 1973*a*, *c*).

A further property of levamisole is its ability to restore immunological reactivity in aged animals and anergic patients (Renoux and Renoux, 1972*b*; Tripodi *et al.*, 1973; Hirshaut *et al.*, 1973; Verhaegen *et al.*, 1973). In this context, Chirigos *et al* (1973) reported that while levamisole alone had no effect on growth of a Moloney virus induced murine leukaemia, marked protective effects were achieved when the drug was administered during BCNU induced tumour remission. This synergistic effect between conventional chemotherapy and levamisole treatment has been interpreted as due to early restoration of cell mediated immunity, destroying remaining tumour cells when at a minimal level (Perk *et al.*, 1975). Similar tests are in progress to assess the combined effects of levamisole and chemotherapeutic agents in the treatment of the rat tumours described in this paper. However, the conclusion from the present studies is that levamisole, while reported to have a broad spectrum of immunological effects, may not necessarily be as effective as other adjuvants if used alone as an immunotherapeutic agent, and further experimental studies may be needed to form a rational basis for a clinical application of the compound.

This work was supported by a grant from the Cancer Research Campaign. We thank Janssen Pharmaceutica, Beerse, Belgium for the supply of levamisole.

### REFERENCES

AMERY, W. (1975) Levamisole. *Lancet*, i, 389.

BALDWIN, R. W. (1966) Tumour Specific Immunity against Spontaneous Rat Tumours. *Int. J. Cancer*, **1**, 257.

BALDWIN, R. W. & PIMM, M. V. (1971) Influence of BCG Infection on Growth of 3-methylcholanthrene-induced Rat Sarcomas. *Eur. J. clin. biol. Res.*, **16**, 875.

BALDWIN, R. W. & PIMM, M. V. (1973*a*) BCG Immunotherapy of Local Subcutaneous Growths and Post-Surgical Pulmonary Metastases of a Transplanted Rat Epithelioma of Spontaneous Origin. *Int. J. Cancer*, **12**, 420.

BALDWIN, R. W. & PIMM, M. V. (1973*b*) BCG Immunotherapy of a Rat Sarcoma. *Br. J. Cancer*, **28**, 281.

BALDWIN, R. W. & PIMM, M. V. (1973*c*) BCG Immunotherapy of Pulmonary Growths from Intravenously Transferred Rat Tumour Cells. *Br. J. Cancer*, **27**, 48.

BALDWIN, R. W., COOK, A. J., HOPPER, D. G. & PIMM, M. V. (1974) Radiation Killed BCG in the Treatment of Transplanted Rat Tumours. *Int. J. Cancer*, **13**, 743.

CHIRIGOS, M. A., PEARSON, J. W. & PRYOR, J. (1973) Augmentation of Chemotherapeutically Induced Remission of a Murine Leukaemia by a Chemical Immunoadjuvant. *Cancer Res.*, **33**, 2615.

FLANNERY, G. R., ROLLAND, J. M. & NAIRN, R. C. (1975) Levamisole. *Lancet*, i, 750.

HIRSHAUT, Y., PINSKY, C., MARQUARDT, H. & OETTGEN, H. F. (1973) Effects of Levamisole on Delayed Hypersensitivity Reactions in Cancer Patients. *Proc. Am. Ass. Cancer Res.*, **14**, 109.

HOEBEKE, J. & FRANCHI, G. (1973) Influence of Tetramisole and Its Optical Isomers on the Mononuclear Phagocytic System: Effect on Carbon Clearance in Mice. *J. Reticuloendothel. Soc.*, **14**, 317.

LAUCIUS, J. F., BODURTHA, A. J., MASTRENGELO, M. J. & CREECH, R. H. (1974) Bacillus Calmette-Guérin in the Treatment of Neoplastic Disease. *J. Reticuloendothel. Soc.*, **16**, 347.

PERK, K., CHIRIGOS, M. A., FUHRMAN, F. & PETTIGREW, H. (1975) Some Aspects of Host Response to Levamisole after Chemotherapy in a Murine Leukemia. *J. natn. Cancer Inst.*, **54**, 253.

PIMM, M. V. & HOPPER, D. G. (1975) Role of Immunocompetence in Localized BCG Suppression of Tumour Growth (Abstract). *Br. J. Cancer*. In the press.

POTTER, C. W., CARR, I., JENNINGS, R., REES, R. C., MCGINTY, F. & RICHARDSON, V. M. (1974) Levamisole Inactive in Treatment of Four Animal Tumours. *Nature, New Biol.*, **249**, 567.

RENOUX, G. & RENOUX, M. (1971) Effet Immunostimulant d'un Imidothiazole dans l'Immunisation des Souris contre l'Infection par Brucella abortus. *C. R. Acad. Sci. Paris* [*D*], **272**, 349.

RENOUX, G. & RENOUX, M. (1972*a*) Action Immunostimulante de dérivés du Phenylimidothiazole sur les Cellules Spleniques Formatrices d'Anticorps. *C. R. Acad. Sci. Paris* [*D*], **274**, 756.

RENOUX, G. & RENOUX, M. (1972*b*) Restauration par le Phenylimidothiazole de le Réponse Immunologique des Souris Âgées. *C. R. Acad. Sci.* [*D*], **274**, 3034.

RENOUX, G. & RENOUX, M. (1972*c*) Levamisole Inhibits and Cures a Solid Malignant Tumour and its Pulmonary Metastases in Mice. *Nature, New Biol.*, **240**, 217.

TRIPODI, D., PARKS, L. C. & BRUGMANS, J. (1973) Drug-induced Restoration of Cutaneous Delayed Hypersensitivity in Anergic Patients with Cancer. *New Engl. J. Med.*, **289**, 354.

VERHAEGEN, H., DE CREE, J., DE COCK, W. & VERBRUGGEN, F. (1973) Levamisole and the Immune Response. *New Engl. J. Med.*, **289**, 1148,

WEBSTER, D. J. T. & HUGHES, L. E. (1975) Levamisole. *Lancet*, i, 389.

WEXLER, H. (1966) Accurate Identification of Experimental Pulmonary Metastases. *J. natn. Cancer Inst.*, **36**, 641.